# The link between non-routine problem solving success levels and strategic flexibility of gifted fourth-grade students

**DOI:** 10.3389/fpsyg.2025.1614829

**Published:** 2025-08-15

**Authors:** Taliha Keleş, Yeliz Yazgan

**Affiliations:** ^1^Mathematics Department, Halil Inalcık Science and Art Center, Ministry of National Education, Bursa, Türkiye; ^2^Elementary Education Department, Education Faculty, Bursa Uludag University, Bursa, Türkiye

**Keywords:** giftedness, non-routine problems, non-routine problem solving, strategic flexibility, binary logistic regression, fourth-grade students

## Abstract

**Introduction:**

The purpose of this correlational study is to explore how success levels in solving non-routine problems among gifted fourth-grade students are linked to their strategic flexibility.

**Methods:**

Data were gathered from 165 gifted fourth-grade students at a Science and Art Center in Bursa/Türkiye. Binary logistic regression was employed to assess the impact of gender and various indicators of strategic flexibility on success levels in non-routine problem solving.

**Results:**

The findings reveal that these students perform exceptionally well in both non-routine problem solving and strategic flexibility. The most commonly used strategies among students are “drawing figures or diagrams”, “reasoning”, and “working backward.” The strategic flexibility indicators with the highest average usage are “selection of the appropriate strategy”, “strategy knowledge”, and “changing strategies when encountering different problems”. A significant and strong correlation was observed between their success in non-routine problem solving and strategic flexibility. Within the regression model, the ability to “select appropriate strategies” emerged as a significant predictor of performance in non-routine problem solving among gifted students.

**Discussion:**

In summary, this study highlights the problem solving strategies used by gifted students in non-routine problems, and the indicators of strategic flexibility that are effective in predicting success.

## 1 Introduction

Strategic flexibility is crucial for solving non-routine problems, which are integral to mathematical proficiency (Hong et al., 2023; Verschaffel, 2024). Non-routine problem solving necessitates complex and higher-order thinking skills (Montague et al., 2014; Niss, 2015), with strategic flexibility encompassing knowledge of multiple strategies (Verschaffel et al., 2009) and the ability to select and apply the most appropriate strategy (Star and Newton, 2009). Fang and Cox (1999) underscore the metacognitive skills involved in selecting and monitoring strategies, as well as adjusting them when necessary. Strategic flexibility is also fundamental for forming a deep and interconnected knowledge base. Research underscores the significance of employing multiple strategies in problem solving (Heinze et al., 2009; Star and Rittle-Johnson, 2008), echoing educational policy documents that prioritize flexibility as a key mathematics learning outcome (e.g., National Council of Teachers of Mathematics, 2000).

Studies in the literature examine strategic flexibility across different subject domains, including algebra (e.g., Jiang et al., 2023; Newton et al., 2010), Fermi problems (Segura and Ferrando, 2023), arithmetic (e.g., Hickendorff, 2018, 2020, 2022; Jóelsdóttir and Andrews, 2023), fraction arithmetic (Silla et al., 2024), addition and subtraction (Lamb et al., 2023), and non-routine problems (Elia et al., 2009; Keleş and Yazgan, 2021). Most research has focused on procedural flexibility, with fewer studies investigating strategic flexibility in non-routine problems (Arslan and Yazgan, 2015; Elia et al., 2009; Keleş and Yazgan, 2021). Lynch and Star (2014) highlight the need for future studies to specifically address strategic flexibility in non-routine problems. Because solving non-routine problems requires individuals to be inclined to shift their cognitive sets or strategies. Furthermore, many studies involve participants from middle school and above. This study aims to comprehensively explore the link between the success of gifted fourth-grade students in solving non-routine problems and their strategic flexibility. Subsequent sections will detail non-routine problem solving, strategic flexibility, their interconnection, and an overview of gifted education in Türkiye.

### 1.1 Theoretical framework

#### 1.1.1 Non-routine problem-solving

Non-routine problems call for use of methods and strategies unfamiliar to the individual. These problems pose a mental challenge because their solutions are not immediately obvious (Polya, 1971). Inoue (2005) defined non-routine problems as those that require the use of unconventional methods and strategies, disrupt cognitive equilibrium when encountered, and challenge students’ thinking processes. The key factor is whether a known rule or algorithm can be applied; hence, what is non-routine for one person might be routine for another. Many resources stress the importance of non-routine problems. For example, the Programme for International Student Assessment (PISA) report highlights “the need for individuals capable of solving non-routine problems in today’s workplaces” (Organisation for Economic Co-operation and Development, 2014, p. 26), noting that about 10 % of workers face non-routine problems daily (Organisation for Economic Co-operation and Development, 2014). The NCTM Standards (2000) also emphasize the importance of exposing students to non-routine problems and developing strategies to solve them. Non-routine problems provide incredible opportunities to demonstrate strategic flexibility compared to algebraic or arithmetic problems (Silla et al., 2024), and allow for multiple solution strategies (Pongsakdi et al., 2020).

The role of solving non-routine problems in supporting students’ metacognitive skills is well-documented in the literature. During the problem-solving process, metacognition refers to an individual’s awareness of their own cognitive processes, as well as their ability to monitor, regulate, and evaluate these processes while solving a problem (Flavell, 1976). Non-routine problems require students to make strategic decisions about the selection and application of strategies, encouraging them to think about the appropriateness and effectiveness of these strategies in the context of the problem (Schoenfeld, 1992; Verschaffel et al., 1999). This process of evaluating and adjusting one’s strategies fosters metacognitive skills, as it requires monitoring, planning, and reflection on key components of metacognition (Goos, 2002). Clearly, metacognition and strategic flexibility are closely linked (Pativisan, 2006).

Most research on non-routine problem solving focuses on evaluating students’ current skills (e.g., Elia et al., 2009), with some experimental studies (e.g., Lee et al., 2014). Other studies investigate behaviors of high- and low-achieving students when solving non-routine problems (e.g., Budak, 2012) or examine gender differences in this area (e.g., Abedalaziz, 2011; Cai, 2002). Additionally, there are studies on the presence of non-routine problems and the strategies used to solve them in mathematics textbooks and curricula (Kolovou et al., 2009; Marchis, 2012; Van Zanten and Van den Heuvel-Panhuizen, 2018). Research on non-routine problems spans from elementary school to university levels and reveals five key findings: (i) Students often find non-routine problems more challenging than routine ones (e.g., Elia et al., 2009). (ii) Providing a framework or program for non-routine problem-solving strategies is beneficial. (iii) Proficient mathematics students are more persistent and can seek alternative methods if their initial approach fails. Teaching strategies directly to low-achieving students can improve success and attitudes towards non-routine problems. (iv) There are mixed results regarding gender differences in non-routine problem solving. (e.g., Abedalaziz, 2011; Cai, 2002; Evans et al., 2021). (v) Non-routine problems are almost absent from mathematics textbooks (e.g., Kolovou et al., 2009; Marchis, 2012).

#### 1.1.2 Strategic flexibility

Recent studies reviewing strategic flexibility (Hong et al., 2023; Verschaffel, 2024) define it as the ability to possess knowledge of multiple strategies and to select the most suitable one(s) from among them. This implies that strategic flexibility is a comprehensive skill involving not just knowledge of strategies, but also the ability to choose and switch between the best strategies. Flexibility in problem solving combines knowledge of multiple strategies with the ability to determine the most effective ones (Liu et al., 2018; Xu et al., 2017). Many studies indicate that, although students know multiple strategies and understand which ones are most appropriate, they do not always choose the most elegant strategy (Newton et al., 2010). To clarify, an appropriate strategy is defined by some researchers as one that maximizes the efficiency and elegance of the solution steps (Coppersmith and Star, 2022). However, the authors of this article define an appropriate strategy as one that best fits the characteristics of the problem and effectively leads to a correct solution (Wang and Star, 2023). While strategic flexibility does not assure an accurate solution, it increases the likelihood of achieving one (De Corte, 2007).

At this point, it would be beneficial to present the procedural flexibility that overlaps with strategic flexibility -the core component of this study -and to elaborate on the distinctions and similarities between them. Procedural flexibility is typically defined as the ability to use multiple procedures for solving a particular type of problem and to choose the most efficient one. It has often been studied in more structured or routine mathematical domains, such as arithmetic or algebra (Rittle-Johnson et al., 2012; Star and Rittle-Johnson, 2008). Procedural flexibility tends to focus on recognizing and applying different known algorithms to solve problems efficiently, without necessarily involving metacognitive strategy shifts. While both procedural and strategy flexibility require knowledge of multiple approaches, strategic flexibility emphasizes higher-order decision making and adaptability across varying problem types, whereas procedural flexibility is more confined to choosing among known procedures within a particular task structure (Hickendorff, 2022; Liu et al., 2018).

There are relatively few studies examining students’ flexibility in non-routine problem solving across different age groups. Elia et al. (2009) investigated the flexibility of 152 high-achieving fourth-grade Dutch students across three non-routine problems. Arslan and Yazgan (2015) studied Turkish students in grades six, seven, and eight across four non-routine problems. Keleş and Yazgan (2021) examined 50 gifted Turkish students in grades eight through eleven across seven non-routine problems. Segura and Ferrando (2023) investigated the strategic flexibility of 224 Spanish prospective teachers across four Fermi problems. Overall, these studies confirm that, regardless of age, strategic flexibility is a crucial skill for problem-solving performance.

#### 1.1.3 The interactions between strategic flexibility, problem solving, and other factors

A few studies have explored the link between strategic flexibility and problem solving. Some research has examined the connection between strategic flexibility and solution accuracy in various mathematical domains (e.g., Elia et al., 2009; Keleş and Yazgan, 2021; Segura and Ferrando, 2023; Star et al., 2022; Torbeyns et al., 2017). Here, we will discuss the findings from three studies on the link between non-routine problem solving and strategic flexibility. Elia et al. (2009) found that students with intra-task strategy flexibility were more successful in reaching correct answers compared to those without such flexibility, though no correlation was found between inter-task strategy flexibility and success. Keleş and Yazgan (2021) reported a substantial and statistically significant correlation of 0.70 between strategy flexibility and success. Segura and Ferrando (2023) also identified a connection between participants’ levels of flexibility and the severity of their errors. Overall, these findings indicate a significant association between flexibility and success in non-routine problem solving.

Studies examining the interactions among strategic flexibility, mathematical success levels, and gender have yielded mixed results. Some research indicates no significant interaction between gender and mathematical success levels (Evans et al., 2021; Wang and Star, 2023). However, other studies suggest variability in the link between flexibility and gender, sometimes favoring females (Star et al., 2015) and other times favoring males (Carr and Jessup, 1997). These findings demonstrate that the results of studies on gender differences in flexibility are inconsistent. Gender can shape individuals’ development of distinct cognitive processes based on societal roles and expectations (Karakuş, 2024). Social norms related to gender may impact how gifted individuals approach non-routine problem-solving. Therefore, exploring how gender influences non-routine problem-solving in gifted students is essential to inform effective educational strategies and approaches in this field.

#### 1.1.4 Giftedness

In the early 20th century, giftedness was predominantly understood and evaluated through intelligence tests (Terman, 1954). However, this approach has evolved into a multidimensional and dynamic perspective that considers environmental factors (Gagné, 2010; Gardner, 1983; Renzulli, 1978; Sternberg and Kaufman, 2018). In his Three-Ring Model, Renzulli (1978) redefined the concept of giftedness, emphasizing that gifted individuals should not only possess high intelligence but also demonstrate characteristics such as creativity and task commitment. Renzulli and Reis (2018) state that this model remains relevant today and continues to influence educational policies. The importance of high intellectual capacity as a key criterion in defining giftedness has been consistently highlighted (Renzulli and Reis, 2018; Sternberg and Kaufman, 2018). In Renzulli’s (1978) Three-Ring Model of giftedness, above-average ability corresponds to above-average mathematical talent, mathematical thinking, the application of mathematical knowledge, and the ability to apply it to different problem situations. Creativity in the model refers to generating new and original solutions to mathematical problems, whereas task commitment represents the focus and perseverance required to work on mathematical problems (Schindler and Rott, 2017).

#### 1.1.5 Gifted programs in Türkiye

In Türkiye, Science and Art Centers (SACs) are institutions established by the state to foster the creativity of gifted students, to instill a scientific study discipline according to their talents, to encourage interdisciplinary thinking, to solve problems, and to contribute to national development (Ministry of National Education, 2024). Students in grades 1, 2, and 3 are first nominated for SACs, then the nominated students undergo a preliminary evaluation, and finally, the students who pass the preliminary evaluation are admitted based on the results of individual assessments (Ministry of National Education, 2023). Intelligence tests are used in individual assessments.

Education and training activities at SAC are conducted individually or in groups outside of regular school hours. Students continue their education at SAC from 2nd grade to 12th grade. Gifted students at SACs complete a total of five programs: adaptation (2nd grade), support education (3rd and 4th grades), recognition of individual talents (5th and 6th grades), development of special talents (7th and 8th grades), and project production and management (9th, 10th, 11th, and 12th grades). At SACs, project-based, interdisciplinary, enriched, and differentiated education programs tailored to students’ talents are implemented, and educational activities are organized to realize original products, projects, and productions (Ministry of National Education, 2024). Furthermore, the educational activities include practices aimed at developing students’ higher-order thinking skills (Ministry of National Education, 2024). The support education, recognition of individual talents, and development of special talents programs include outcomes aimed at improving students’ problem-solving skills (Ministry of National Education, 2019). The implementation of these outcomes is not mandatory for all students. Classroom/subject teachers can differentiate and enrich the program in a student-centered and interdisciplinary manner, taking into account students’ interests, talents, and potentials to enable them to acquire higher-order mental, personal, and academic skills such as problem-solving and creativity (Ministry of National Education, 2024). This indicates that SAC teachers can take the initiative in the selection and implementation of the program.

#### 1.1.6 The current study and research questions

While previous research has examined the relationship between strategic flexibility and non-routine problem-solving among gifted students, much of this work has predominantly focused on high school students (Keleş and Yazgan, 2021, 2022). These studies often explored strategic flexibility through inter-task and intra-task strategic flexibility, emphasizing older students’ cognitive strategies across different problem contexts. However, investigations targeting younger gifted students remain notably limited. In particular, few studies have systematically explored how strategic flexibility contributes to non-routine problem-solving success among elementary school students (Elia et al., 2009). Given that fourth grade marks a pivotal period in the development of complex cognitive and metacognitive skills, understanding how gifted learners at this stage employ strategic flexibility is critical. Early identification of effective strategic behaviors may not only inform enrichment and differentiated instruction but also support the cultivation of advanced problem-solving abilities over time. Moreover, although the general concept of strategic flexibility has been addressed in earlier studies, there is a lack of empirical research that dissects specific indicators of strategic flexibility (e.g., such as strategy knowledge, changing strategies when encountering different problems) particularly in relation to non-routine problem-solving performance at the elementary level. Non-routine problems contribute to the development of higher-order thinking skills such as creativity, analysis, and synthesis (Cai and Lester, 2005; Schoenfeld, 2013). Considering that gifted students possess high intellectual abilities and creative potential (National Association for Gifted Children, 2009), it is deemed valuable to uncover the factors that trigger and influence the problem-solving success levels of gifted students. Numerous researchers have emphasized the importance of cultivating a systematic problem-solving approach in students (e.g., Schoenfeld, 1985). Consequently, examining fourth-grade students’ strategic flexibility and performance on non-routine problems holds a significant value. This study seeks to address this significant gap by investigating the relationship between strategic flexibility indicators and non-routine problem-solving success among gifted fourth-grade students. By identifying the strategic flexibility components that most strongly predict success, this research contributes to a more nuanced understanding of early cognitive development in gifted populations and offers evidence-based insights for optimizing educational practices aimed at fostering flexibility and problem-solving expertise from an early age. This study aims to examine the extent to which gender and strategic flexibility indicator scores serve as determinants of students’ high or low success levels in solving non-routine problems. In this context, the current research addresses three questions: (1) What are the levels of success and strategic flexibility in solving non-routine problems among gifted fourth-grade students? At SACs, the aim is to develop problem solving, critical and creative thinking, effective decision-making, and other skills of gifted students identified in the field of general mental ability and attending support education programs (Ministry of National Education, 2024). Therefore, it is expected that students’ success in solving non-routine problems and their levels of strategic flexibility would be above average. (2) Is there a significant link between gifted fourth-grade students’ success in solving non-routine problems and their strategic flexibility? The literature presents mixed evidence regarding the relationship between strategic flexibility and accuracy in problem-solving. While some studies suggest a relationship between flexibility and accuracy in solving linear equations (e.g., Star et al., 2022), there is very little evidence supporting the relationship between strategic flexibility and non-routine problems (e.g., Keleş and Yazgan, 2021). We hypothesize that there will be a significant link between students’ success in solving non-routine problems and their strategic flexibility. (3) Which factors, gender and strategic flexibility indicators, affect the success levels of gifted fourth-grade students in solving non-routine problems? Gender can influence opportunities for learning mathematics (Byrnes and Wasik, 2009). For example, Star et al. (2015) referred to a notable relationship between gender and flexibility, favoring girls. Additionally, Keleş and Yazgan’s (2022) study showed that strategic flexibility indicators play a key role in the success of solving non-routine problems. Therefore, we hypothesize that both gender and strategic flexibility indicators will influence the success of solving non-routine problems.

## 2 Materials and methods

### 2.1 Research design

This study is designed in a correlational survey model as it aims to identify the factors affecting the success of gifted fourth-grade students in solving non-routine problems. Correlational surveys are utilized to explore the relationships between two or more variables and to provide a descriptive analysis of the current state (Tabachnick and Fidell, 2013).

### 2.2 Participants

The sample of the study consisted of 174 fourth-grade students, aged 9–10, enrolled in a support program at a SAC in Bursa, located in western Türkiye. However, after removing outliers, the remaining 165 students (97 males, 68 females) constituted the participants of the study. Students were coded as S1, S2, S3,…, S165. Participants were selected voluntarily, and assurances were given that their responses would be kept confidential. Ethical committee approvals were obtained before the data collection process.

### 2.3 Data collection instrument

To assess the non-routine problem-solving success and strategic flexibility of gifted students, we used six non-routine problems (see Appendix). The problems were adapted from various sources in the literature (Herr and Johnson, 2002; Lee, 1982; Posamentier and Krulik, 2009). After completing the problem-solving test, interviews based on the stimulated recall technique were conducted with students. The Stimulated Recall (SR) technique, introduced by Calderhead (1981), is a method used to help individuals recall thought processes and strategies during problem-solving. In this study, SR was employed as a tool to collect the necessary data, in which students were shown their solution sheets and asked to provide explanations about their problem-solving processes. This approach enabled the collection of in-depth data on the students’ solution strategies.

### 2.4 Data collection process

#### 2.4.1 Implementation

**Figure 1:**
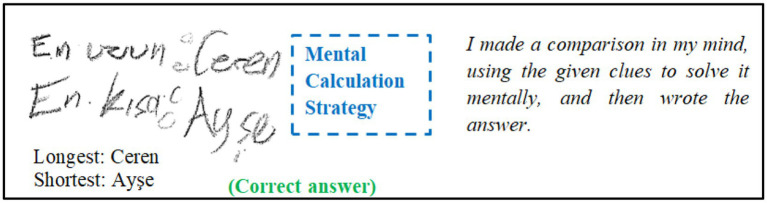
S12’s solution (correct) for Problem 6 regarding the “mental calculation strategy”.

The data were gathered by the first author, who also serves as a mathematics teacher at the SAC. The data collection process occurred in three phases. Initially, six problems were presented to students, each on individual sheets of paper, and they were instructed to provide detailed solutions on answer sheets. Subsequently, in the second phase, students were tasked with re-solving each problem using as many diverse strategies as possible, without altering or supplementing their initial solutions from the first phase. The objective of the second phase was to assess each student’s proficiency in employing multiple strategies for each problem. Additionally, students were instructed not to use erasers during both stages and were required to provide detailed solutions to the best of their ability. The test lasted approximately 80 min. Data were collected from 21 different groups, each consisting of at least seven to ten students, and the process took approximately 4 weeks. In the third stage, immediately after completing the problem-solving test, individual interviews lasting approximately 10–15 min were conducted with each student using the SR technique. During the interviews, participants were asked questions such as “How did you solve the problem?,” “How did you arrive at your solution?,” and “Can you explain your solution?” The primary aim of these interviews was to uncover the strategies that students used in their solutions. While conducting the interviews, the researcher took notes on the backs of the students’ answer sheets. For instance, while the strategies used by some students could easily be inferred from their responses, it was challenging to determine the strategies employed by others based solely on their solutions. At this point, the individual interviews provided clearer insights into the strategies used. For example, in the case of S12’s response to Problem 6 (Figure 1), it was observed that the student wrote the answer directly without performing any mathematical calculations or drawings. During the interview, the question “How did you solve problem 6?” was asked. As can be seen from the solution in Figure 1 and the interview excerpt, S12 solved the problem with the “mental calculation strategy.” Based on this explanation, the strategy used by the student for this question was classified as the “mental calculation.”

### 2.5 Data coding

Data coding was conducted in three stages. Firstly, accuracy was determined and coded solely based on responses given during the first stage. Two points were coded for correct answers, one point for partially correct answers, and zero points for incorrect or blank answers when evaluating non-routine problems. The maximum score attainable was 12 points, reflecting the student’s score in non-routine problem solving. The reliability of the non-routine problem-solving test was assessed using Cronbach’s alpha, resulting in a coefficient of *α* = 0.67.

**Figure 2:**
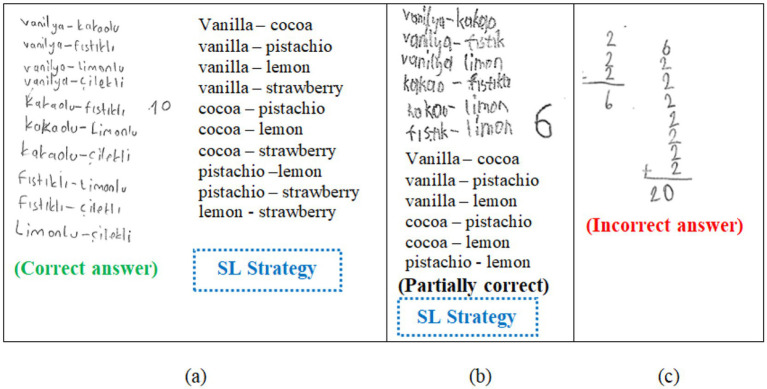
Sample solutions and strategies for Problem 2.

Secondly, the strategies used by students were systematically coded according to the non-routine problem-solving strategies commonly referenced in academic literature (Herr and Johnson, 2002; Posamentier and Krulik, 2008, 2009). Upon reviewing student papers, it was observed that strategies such as “working backward” (WB), “systematic listing” (SL), “looking for a pattern” (LP), “drawing figures or diagrams” (DD), “guessing and checking” (GC), “reasoning” (R), “creating tables” (CT), “assigning numerical values” (ANV), “mental calculation” (MC), and “writing an equation or inequality” (WEI) were utilized. If a strategy was employed in solving the problem, one point was awarded, and if not, zero points were given. To be scored as one point, it was assessed whether the strategy was appropriate for the solution and contributed to it, regardless of whether the problem was solved correctly. For example, Figure 2 represents the sample coding for Problem 2 regarding accuracy and strategy.

In Figure 2a, the student with code S71 systematically listed all possible situations in problem 2 and reached the correct answer 10. Since this student’s solution was completely solved correctly, two points were given, and one point was given for the “systematic listing” in terms of strategy. In Figure 2b, a student with code S5 skipped four possible situations in Problem 2 and reached six instead of 10 as the answer. This solution was evaluated as partially correct, and the student was given one point for correctness and one point for the “systematic listing” in terms of strategy. In Figure 2c, the student with code S68 listed the ice cream varieties in Problem 2, although the order was not important, paying attention to the order reached a result of 20. This student’s solution was evaluated as incorrect and was given zero points for correctness; since this student could not use a strategy, this student also received zero points for strategy.

**Figure 3:**
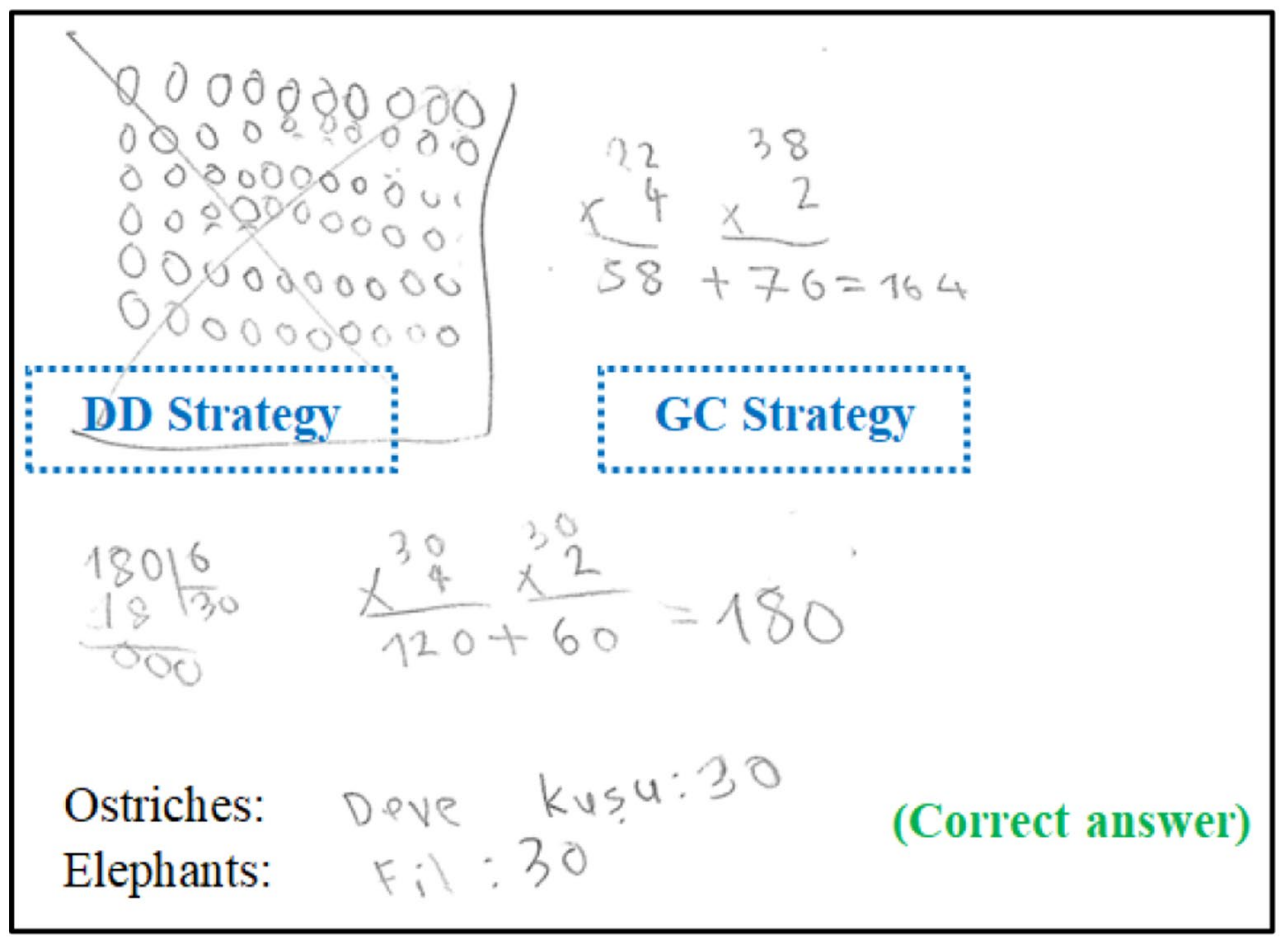
An example of a solution related to the indicator, “changing the strategy when it did not work”.

Thirdly, for the assessment of strategic flexibility, strategic flexibility indicators consisting of seven indicators identified by Keleş and Yazgan (2022) were used. These indicators are “strategy knowledge,” “selection of the appropriate strategy,” “changing the strategy when it did not work,” “after solving the problem, solving it again with a different strategy,” “ability to use several strategies simultaneously for solving a problem,” “checking the correctness of the solution with a different strategy,” and “changing strategies when encountering different problems”. An Excel spreadsheet was created to code these indicators, and the frequency of each student’s demonstration of these indicators was calculated. For example, “strategy knowledge” measured how many different strategies the student used across all problem solutions. “Selection of the appropriate strategy” measured how often the student selected appropriate strategies for solving the problems. Both the responses given during the first stage and the second stage were coded when coding strategies and strategic indicators. The student’s strategic flexibility score was total of calculated by summing the scores of the strategic flexibility indicators. The reliability of the strategic flexibility indicator scores was assessed using Cronbach’s alpha, resulting in a coefficient of *α* = 0.79. The results of the strategic flexibility score are shown in Table 1. For example, Figure 3 presents an example of a student’s solution demonstrating the indicator “changing the strategy when it did not work”.

**Table 1:** Distribution of strategic flexibility indicator scores.

Scores	Strategy knowledge	Selection of the appropriate strategy	Changing the strategy when it did not work	After solving the problem, solving it again with a different strategy	Ability to use several strategies simultaneously for solving a problem	Checking the correctness of the solution with a different strategy	Changing strategies when encountering different problems
0	–	–	158 (95.8%)	116 (70.3%)	123 (74.5%)	165 (100%)	–
1	–	–	6 (3.6%)	32 (19.4%)	41 (24.8%)	–	–
2	8 (4.8%)	3 (1.8%)	1 (0.6%)	15 (9.1)	2 (0.6%)	–	8 (4.8%)
3	25 (15.2%)	20 (12.1%)	–	2 (1.2%)	–	–	28 (17%)
4	48 (29.1%)	46 (27.9%)	–	–	–	–	52 (31.5%)
5	39 (23.6%)	49 (29.7%)	–	–	–	–	47 (28.5%)
6	32 (19.4%)	46 (27.9%)	–	–	–	–	30 (18.2%)
7	9 (5.5%)	1 (0.6%)	–	–	–	–	–
8	4 (2.4%)	–	–	–	–	–	–

When S42’s solution to the fourth problem is examined, it is seen that the student first applied the strategy of drawing shapes, but then gave up this method by crossing it out. He then reached the correct solution using the “guessing and checking” strategy.

**Figure 4:**
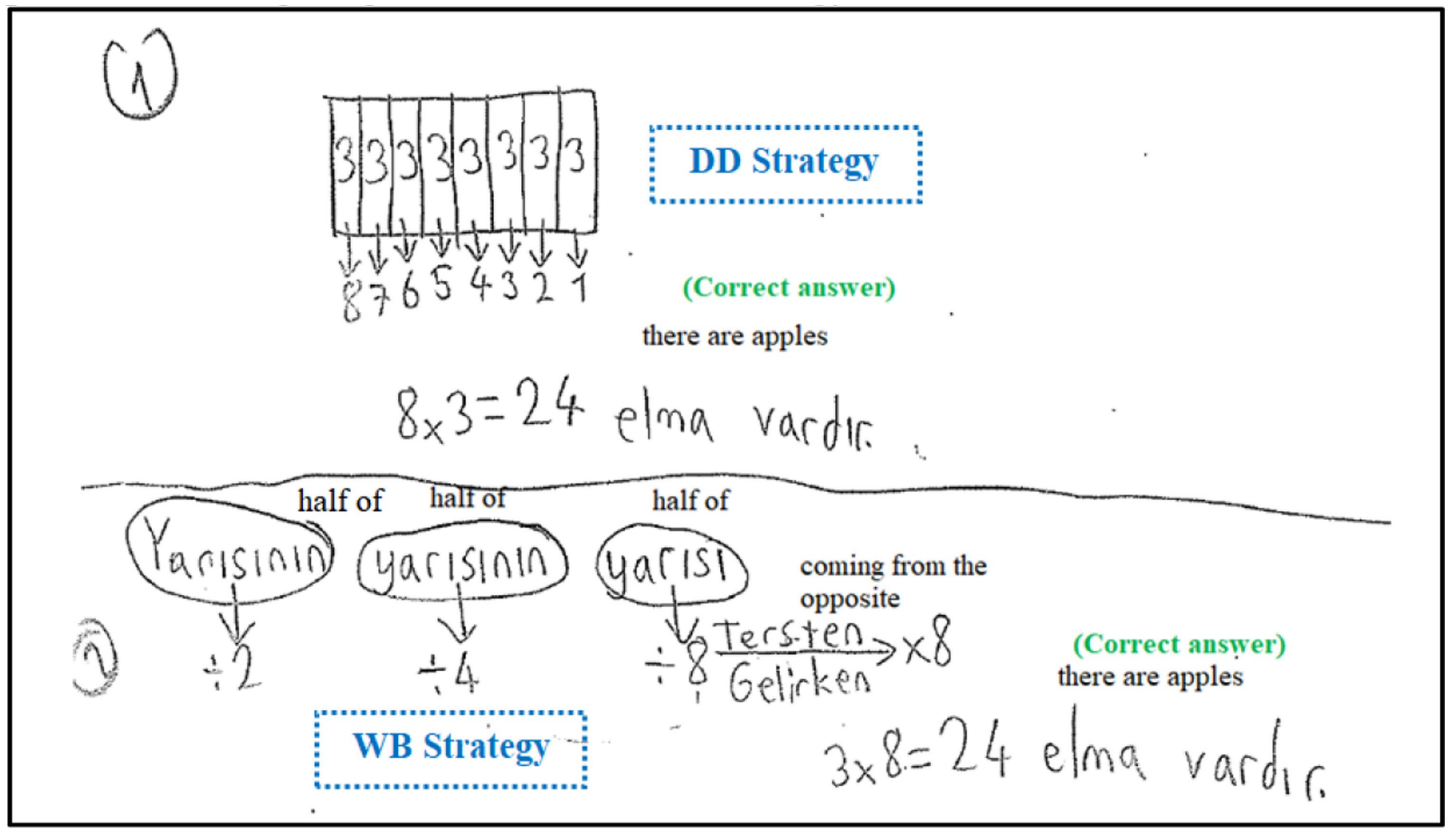
An example of a solution related to the indicator, “after solving the problem, solving it again with a different strategy”.

Figure 4 presents an example of a student’s solution demonstrating the indicator “after solving the problem, solving it again with a different strategy”.

When S46’s solution to the first problem is examined, the student solved the problem correctly by using the “drawing figures or diagrams” strategy as the first way. It is seen that student solved the problem correctly by using the “working backward” strategy as the second way.

**Figure 5:**
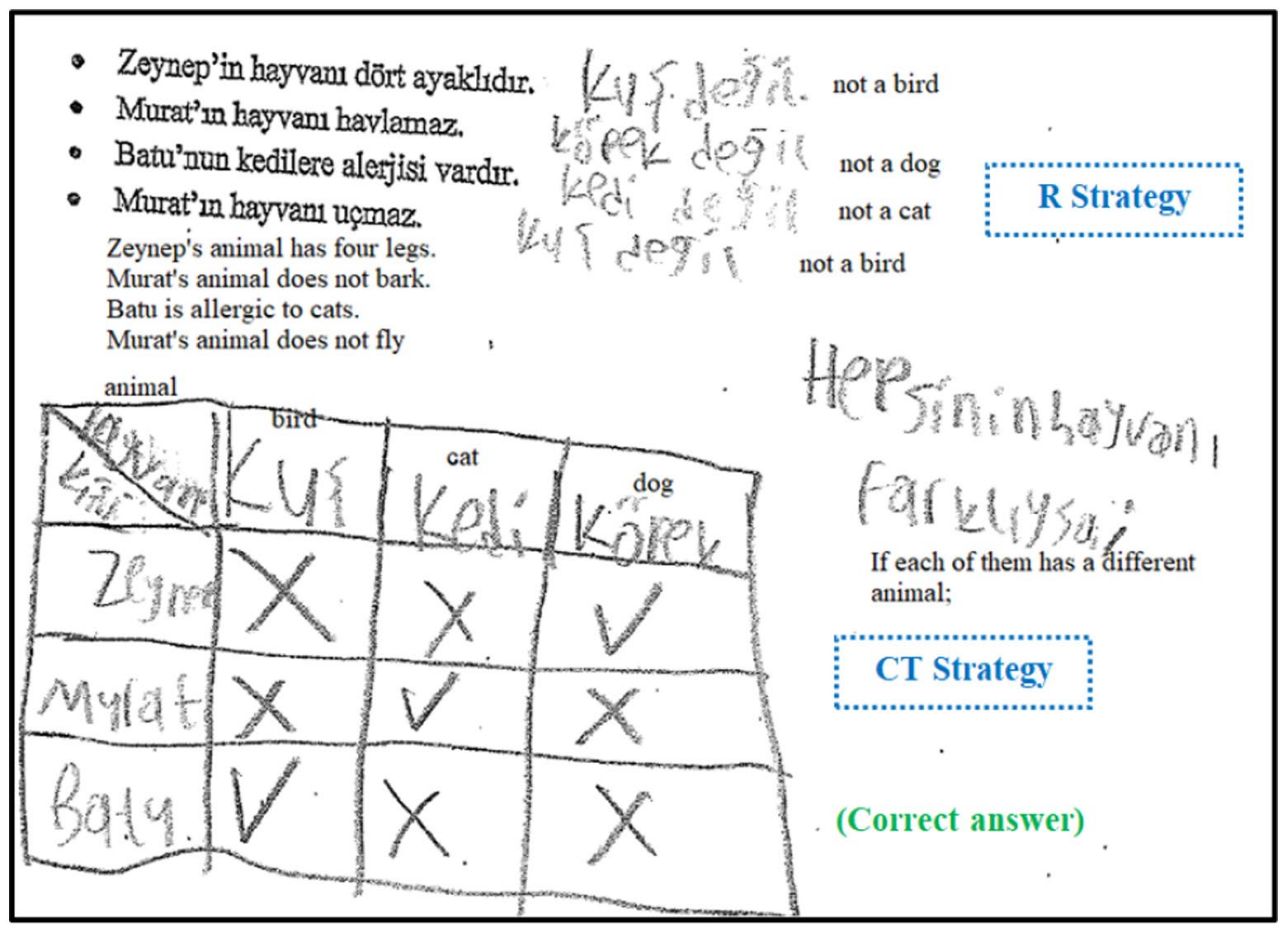
An example of a solution related to the indicator, “ability to use several strategies simultaneously for solving a problem”.

Figure 5 presents an example of a student’s solution demonstrating the indicator “ability to use several strategies simultaneously for solving a problem”.

In Figure 5, when S13’s solution to the fifth problem is examined, it is seen that he used “reasoning” and “creating tables” strategies together to solve the problem.

**Figure 6:**
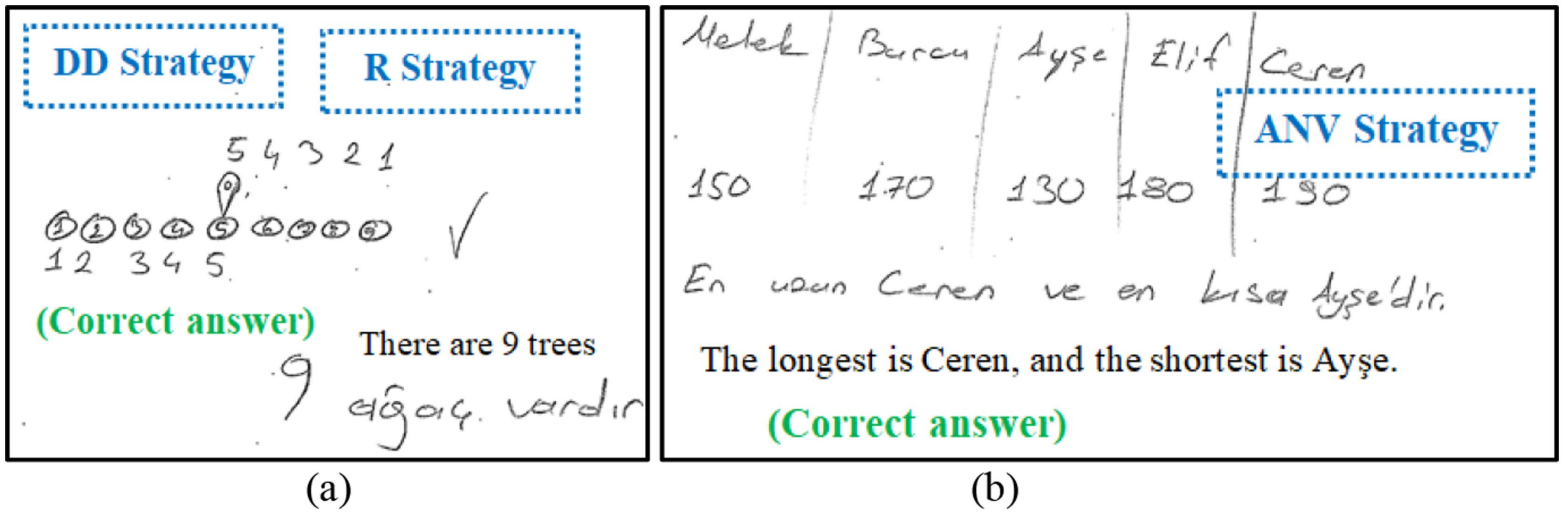
Example of a student’s (correct) solutions related to the indicator “changing strategies when encountering different problems”.

Figure 6 presents an example of a student’s solution demonstrating the indicator “changing strategies when encountering different problems”.

In Figure 6, only the answers given by S110 to the third and sixth problems can be seen. While she used the “drawing figures or diagrams” and “reasoning” strategies for the third problem (Figure 6a), she easily switched to the “assigning numerical values” strategy for the sixth problem (Figure 6b).

The solutions provided by gifted students for non-routine problems were independently coded by two researchers, focusing on accuracy and indicators of strategic flexibility. To ensure inter-rater reliability, Cohen’s Kappa was calculated, yielding values ranging from 0.73 to 0.89 for accuracy across the items and from 0.79 to 1 for indicators of strategic flexibility. To resolve inconsistencies between different codes, the researchers discussed and reached a consensus.

### 2.6 Data analysis

We utilized SPSS 26 software for the data analysis. In logistic regression, we conducted an outlier check, which is an assumption for the independent variables. We compared the Mahalanobis distance values of the independent variables with the chi-square value and excluded data from nine students from the dataset.

In this study, data analysis proceeded through three stages. First, descriptive statistics were employed to assess students’ success in solving non-routine problems and their strategic flexibility. Second, the Pearson Product–Moment Correlation Coefficient (r) was computed to investigate the link between non-routine problem solving success and strategic flexibility scores. Third, a binary logistic regression analysis was conducted to determine which factors, including gender and strategic flexibility indicators, influenced students’ success levels in solving non-routine problems. Gender and strategic flexibility indicators were treated as independent variables, while the level of non-routine problem-solving success served as the dependent variable.

The overall mean score for problem-solving was 8.5. Scores between 0 and 8 were classified as low success, while scores of 9 or above were classified as high success. Among the participants, 77 students (46.7%) fell into the low success category, while 88 students (53.3%) fell into the high success category. A significant difference in mean scores for non-routine problem-solving was observed between the low success and high success groups (t = −19.1, *p* < 0.05).

To meet the assumptions of logistic regression analysis, multicollinearity among the independent variables was initially assessed. Correlations among the independent variables were examined, with a correlation value above 0.80 indicating potential multicollinearity and a value above 0.90 suggesting serious multicollinearity (Field, 2009). Additionally, tolerance values (TV) greater than 0.10, variance inflation factor (VIF) values less than 10, and condition index (CI) values below 30 were examined to ensure no multicollinearity issues (Tabachnick and Fidell, 2013). Due to a high correlation (r = 0.91) between the “strategy knowledge” variable and the variable “changing strategies when encountering different problems” (see Table 2), the “strategy knowledge” variable was removed from the model. Furthermore, the variable “checking the correctness of the solution with a different strategy” was also removed from the model due to lack of data.

**Table 2:** Correlations between variables.

Variables	1	2	3	4	5	6	7	8
1. Non-routine problem-solving scores	1							
2. Strategy knowledge	0.70**	1						
3. Selection of the appropriate strategy	0.86**	0.80**	1					
4. Changing the strategy when it did not work	0.17*	0.14	0.19*	1				
5. After solving the problem, solving it again with a different strategy	0.32**	0.57**	0.35**	−0.01	1			
6. Ability to use several strategies simultaneously for solving a problem	0.13	0.10	0.14	−0.06	−0.05	1		
7. Changing strategies when encountering different problems	0.76**	0.91**	0.88**	0.15*	0.38**	0.10	1	
8. Strategic flexibility scores	0.79**	0.96**	0.90**	0.20**	0.58**	0.20**	0.94**	1

*p < 0.05, **p < 0.01.

In the examined dataset, the TV values were greater than 0.10, the VIF values were less than 10, and the CI values were less than 30, indicating the absence of multicollinearity problems among the independent variables. With the assumptions for binary logistic regression analysis satisfied, the analysis proceeded accordingly.

Logistic regression analysis serves as a method for classifying outcomes by estimating the probability of the dependent variable based on the independent variables (Tabachnick and Fidell, 2013). The main objective is to develop a model that most accurately represents the relationship between the dependent variable and the independent variables (Hosmer et al., 1997). In binary logistic regression, the model calculates the probability that the dependent variable will fall into one of two categories for a given observation (Field, 2009). Using the enter method with data from 165 students, a binary logistic regression model was employed as follows (see Equation 1):


(1) $$P(Y)=\frac{e^{z}}{1+e^{z}}=\frac{1}{1+e^{-z}}$$

Here, P(Y) represents the probability of event Y occurring, where e is the base of the natural logarithm (Field, 2009; Tabachnick and Fidell, 2013). The variable Z signifies the aggregate impact of all independent variables included in the model and is defined as follows (see Equation 2):


(2) $$Z=\beta_{0}+\beta_{1}X_{1}+\beta_{2}X_{2}+\ldots +\beta_{P}X_{P}$$

$\beta_{0},\beta_{1},\beta_{2},\ldots \beta_{P}$ are the coefficients of the logistic regression. The computation of these coefficients is outlined as follows (see Equation 3).


(3) $$\mathrm{Ln}(\frac{P(Y)}{Q(Y)})=\beta_{0}+\beta_{1}X_{1}+\beta_{2}X_{2}+\ldots +\beta_{P}X_{P}$$

Here, P(Y) denotes the probability of the event occurring, Q(Y) represents the probability of the event not occurring, and L_n_ denotes the natural logarithm.

## 3 Results

In this section, the findings are presented sequentially based on the research problems.

### 3.1 Success and strategic flexibility levels in solving non-routine problems

Table 3 shows the percentage distribution of students’ responses to the problems.

**Table 3:** Percentage distribution of responses to the problems.

Response type	Correct		Partial correct		Incorrect/ Blank	
	*N*	%	*N*	%	*N*	%
Problem 1	93	56.4	55	33.3	17	10.3
Problem 2	78	47.3	11	6.7	76	46.1
Problem 3	123	74.5	-	-	42	25.5
Problem 4	77	46.7	1	0.6	87	52.7
Problem 5	153	92.7	-	-	12	7.3
Problem 6	143	86.7	14	8.5	8	4.8
Total	667	67.3	81	8.2	242	24.5

*N*, number of participants.

Upon reviewing Table 3, it is evident that the fifth problem had nearly universal correct responses from students, followed closely by the sixth and third problems. In contrast, the fourth and second problems showed the lowest success rates.

Table 4 presents both the count and percentage of strategies employed by students in tackling non-routine problems. Students often utilized multiple strategies for individual problems, and certain strategies were applied across multiple problems.

**Table 4:** Distribution of strategies used by students.

Strategy	WB	SL	LP	DD	GC	R	CT	ANV	MC	WEI
f	142	73	14	153	90	151	9	6	73	54
%	86.1	44.2	8.5	92.7	54.5	91.5	5.5	3.6	44.2	32.7

WB, working backward; SL, systematic listing; LP, looking for a pattern; DD, drawing figures or diagrams; GC, guessing and checking; R, reasoning; CT, creating tables; ANV, assigning numerical values; MC, mental calculation; WEI, writing an equation or inequality.

As seen in Table 4, from the perspective of strategy usage, the strategies of “drawing figures or diagrams” (92.7%), “reasoning” (91.5%), and “working backward” (86.1%) were the most frequently used. Conversely, the strategies of “looking for a pattern” (8.5%), “creating tables” (5.5%) and “assigning numerical values” (3.6%) were the least used. Table 1 shows the findings related to students’ strategic flexibility indicator scores.

Descriptive statistics of students’ strategic flexibility indicator scores are presented in Table 5.

**Table 5:** Descriptive statistics of strategic flexibility indicator scores.

Strategic flexibility indicator	Strategy knowledge	Selection of the appropriate strategy	Changing the strategy when it did not work	After solving the problem, solving it again with a different strategy	Ability to use several strategies simultaneously for solving a problem	Checking the correctness of the solution with a different strategy	Changing strategies when encountering different problems
Mean	4.63	4.71	0.04	0.41	0.26	0	4.38
St. Dev.	1.35	1.07	0.24	0.70	0.45	0	1.11
Skewness	0.21	−0.33	5.47	1.62	1.29	0	−0.20
Kurtosis	−0.30	−0.68	32.70	1.81	0.21	0	−0.68
Minimum	2	2	0	0	0	0	2
Maximum	8	7	2	3	2	0	6

When examining Tables 1, 5, it is observed that the indicators with the highest averages are, respectively, “selection of the appropriate strategy,” “strategy knowledge,” and “changing strategies when encountering different problems”. The averages of usage for the other indicators are relatively low. The indicator “changing the strategy when it does not work” is the least demonstrated. Additionally, it was found that the indicator “checking the correctness of the solution with a different strategy” was not used at all.

Descriptive statistics for students’ non-routine problem solving scores and strategic flexibility scores are presented in Table 6.

**Table 6:** Descriptive statistics for non-routine problem solving and strategic flexibility scores.

Scores	N	Mean	St. Dev.	Skewness	Kurtosis	Min.	Max.	Sum
Non-routine problem solving	165	8.57	2.24	−0.24	−0.54	3	12	1,415
Strategic flexibility	165	14.45	3.88	0	−0.65	6	23	2,385

The averages of students’ non-routine problem-solving scores and strategic flexibility scores were calculated as 8.57 and 14.45, respectively (Table 6). Considering that students could score a maximum of 12 in non-routine problem solving and a maximum of 23 from the total of the indicators, it is observed that their performance in both non-routine problem solving and strategic flexibility is above average.

### 3.2 The link between success in non-routine problem-solving and strategic flexibility

Correlations between variables are shown in Table 2.

As seen in Table 2, the highest, positive, and significant correlation values between the non-routine problem solving and the indicator scores are for “selection of the appropriate strategy” (r = 0.86) and “changing strategies when encountering different problems” (r = 0.76). The lowest correlation is for the indicator “ability to use several strategies simultaneously for solving a problem” (r = 0.13). When examining the correlations among the indicators themselves, the highest significant correlation is between “strategy knowledge” and “changing strategies when encountering different problems” (r = 0.91), while the lowest correlation is between “changing the strategy when it did not work” and “after solving the problem, solving it again with a different strategy” (r = −0.01).

Overall, there is a significantly positive link between the non-routine problem solving and strategic flexibility scores of gifted students (r = 0.79, *p* < 0.01). The highest, positive, and significant correlation values between students’ strategic flexibility and the indicator scores are, respectively, for “strategy knowledge” (r = 0.96), “changing strategies when encountering different problems” (r = 0.94), and “selection of the appropriate strategy” (r = 0.90). The lowest correlations are for “changing the strategy when it did not work” (r = 0.20) and “ability to use several strategies simultaneously for solving a problem” (r = 0.20).

### 3.3 The effects of gender and strategic flexibility indicators on success in solving non-routine problems

The analyses were performed with the inclusion of independent variables in the model. The outcomes of the model summary generated using the Enter method are depicted in Table 7.

**Table 7:** Logistic regression analysis results model fit indices.

Omnibus tests of model coefficients				
		Chi-Square	df	Significance
Step 1	Step	157.23	6	0.00
	Block	157.23	6	0.00
	Model	157.23	6	0.00

Model summary				
		−2 Log likelihood	Cox and Snell R^2^	Nagelkerke R^2^
Step 1		70.76	0.61	0.82

Hosmer and Lemeshow test				
		Chi-Square	df	Significance
Step 1		15.17	8	0.056

When referring to Table 7, the Omnibus Tests of Model Coefficients reveal a Chi-Square value of 157.237, with six degrees of freedom, and a significance level of 0.000 (*p* < 0.05). The *p*-value’s significance for the model chi-square suggests a relationship between the dependent variable and the independent variables. Examining the “model summary” section at the bottom of Table 7, the −2 Log likelihood statistic is recorded as 70.768. This value was compared with the −2 Log likelihood of the initial model and found to be statistically significant. The Cox-Snell R^2^ and Nagelkerke R^2^ values indicate the proportion of variance in the dependent variable accounted for by the model (Field, 2009). The independent variables explain 61.4% of the total variance in the dependent variable according to Cox-Snell R^2^ and 82% according to Nagelkerke R^2^. The Hosmer and Lemeshow test (Chi-Square = 15.173; *p* > 0.05) shows that the model has an adequate level of data fit. The classification table is another indicator of model fit. The binary logistic regression model classification status is presented in Table 8.

**Table 8:** Classification table.

Observed			Predicted		
			Problem solving success level		Percentage correct
			High	Low	
Step 1	Problem solving success level	High	87	1	98.9
		Low	9	68	88.3
	Overall percentage				93.9

When examining the classification status of the logistic regression model given in Table 8, out of 88 students with high problem solving skills, 87 were correctly classified, resulting in an approximate accuracy rate of 99%. For students with low problem solving skills, out of 77 students, 68 were correctly classified, resulting in an approximate accuracy rate of 88%. The overall correct classification rate for the intended model is approximately 94%, indicating that the model performs very well.

The analysis results for the regression coefficients (B), the standard error (S. E.), the Wald statistic (for statistical significance testing), and the odds ratio (Exp (B)) estimates for the intended model variables are provided in Table 9.

**Table 9:** Statistical significance of the variables in the model.

Model variables	B	S.E.	Wald	Sig.	Exp(B)
Gender	0.80	0.67	1.44	0.22	2.24
Selection of the appropriate strategy	3.84	0.87	19.45	0.00	46.60
Changing the strategy when it did not work	18.46	11985.04	0.00	0.99	104401493.94
After solving the problem, solving it again with a different strategy	0.06	0.53	0.013	0.90	1.06
Ability to use several strategies simultaneously for solving a problem	0.72	0.73	0.98	0.32	2.07
Changing strategies when encountering different problems	0.05	0.64	0.008	0.93	1.05
Constant	−18.82	3.24	33.65	0.00	0.00

When examining Table 9, it is observed that the variable “selection of the appropriate strategy” is a significant predictor of non-routine problem solving success. However, gender and other variables are not significant predictors of the dependent variable. According to these results, the predictor variable “selection of the appropriate strategy” increases problem solving success level by a factor of 46.607. This means that an increase of one unit in the predictor variable “selection of the appropriate strategy” leads to a [(1–46.60)0.100] increase in the odds of success in problem solving, approximately 4560.7%. The results indicate that two variables, namely “selection of the appropriate strategy” and the constant term, are significant. Thus, the equation obtained from the model is shown below.


$$\begin{array}{l} \text{Problem solving success level}=\\ 3.842*\text{Selection of the appropriate strategy}-18.824. \end{array}$$

## 4 Discussion

Our initial research question investigated the proficiency of fourth-grade students classified as gifted in tackling non-routine problems, alongside their levels of strategic flexibility. First of all, it has been observed that the most commonly used strategies are “drawing figures or diagrams,” “reasoning,” and “working backward”. This is consistent with previous studies examining the strategic flexibility of gifted students (e.g., Keleş and Yazgan, 2021). These strategies are those in which students demonstrate the highest success in solving non-routine problems. Most students performed well on Problems 6, 5, and 1, respectively. This can be attributed to the appropriateness of strategies such as “drawing figures or diagrams,” “reasoning,” and “working backward” working backward to solve these problems, as well as to the students’ greater experience in employing these strategies. However, most students struggled with Problem 4. This difficulty is thought to be due to the necessity of using the “guess and check” strategy, which requires higher-order cognitive skills to solve this problem.

Upon reviewing the outcomes detailed in Table 6, we observe that students perform above average in achieving correct solutions to non-routine problems and in strategic flexibility. Considering that the students are deemed gifted, this situation can be attributed to their high mathematical proficiency. Results shown in Tables 1, 5 indicate that the averages for “selection of the appropriate strategy,” “strategy knowledge,” and “changing strategies when encountering different questions” are highest in strategic flexibility. This finding aligns with previous studies (e.g., Durkin et al., 2023; Goos et al., 2000; Newton et al., 2020; Star et al., 2022). Moreover, this finding supports the results of previous research indicating that in order to be a flexible problem solver, one must also have various “strategy knowledge” and “selection of the appropriate strategy” skills (Newton et al., 2020; Xu et al., 2017). Our findings suggest that teachers with significant autonomy in differentiating and enriching instruction, considering students’ interests, abilities, and potentials (Ministry of National Education, 2024), may explain the inclusion of non-routine problems in SACs.

Some previous studies (Hickendorff, 2020; Jóelsdóttir and Andrews, 2023) have indicated lower levels of strategic flexibility among young age groups like third and fourth graders. This inconsistency may stem from these studies focusing on different areas of mathematics such as equation solving, algebra problems, fraction arithmetic, and verbal problems. Additionally, we found that the indicator “changing strategies when they do not work,” “ability to use several strategies simultaneously for solving a problem,” and “after solving the problem, solving it again with a different strategy” are the least used, and “checking the accuracy of the solution with a different strategy” is not used at all. This finding contrasts with Pativisan’s (2006) results. Given that students perform above average in achieving correct solutions and their chosen strategies are appropriate, the low occurrence of “changing strategies when they do not work” is expected. However, the complete absence of “checking the accuracy of the solution with a different strategy” may suggest students’ inclination to believe that each problem has only one correct answer and one correct solution method (Schoenfeld, 1992). This situation reveals that most of the students are not able to show the tendency of “changing strategies when they do not work,” “ability to use several strategies simultaneously for solving a problem,” and “checking the accuracy of the solution with a different strategy”. Elia et al. (2009) and Keleş and Yazgan (2021) also reached similar conclusions.

Specifically, the abovementioned belief that every problem has a single correct solution method and one right answer discourages students from exploring alternative strategies after reaching an initial solution (Higgins, 1997; Schoenfeld, 1992). This belief may limit students’ inclination to verify their answers using different approaches, even in the context of non-routine problems where such behavior is especially valuable. As Pativisan (2006) and Star and Rittle-Johnson (2008) have noted, strategic flexibility requires not only procedural knowledge but also a disposition toward monitoring and adjusting one’s approach—a disposition that may be underdeveloped in younger students or in educational cultures where correctness is prioritized over process. In our study, although students demonstrated above-average success and frequently selected appropriate strategies, the lack of engagement with alternative-verification strategies (e.g., checking the correctness of the solution with a different strategy) may reflect a performance-oriented mindset rather than an exploratory one.

Our second research questions investigated the correlation between students’ proficiency in solving non-routine problems and their strategic flexibility. In our study, we observed a significant and strong correlation between success in solving non-routine problems and strategic flexibility. These findings align with previous research on equation problems (Coppersmith and Star, 2022; Star et al., 2022), Fermi problems (Segura and Ferrando, 2023), arithmetic problems (Hickendorff, 2022; Jóelsdóttir and Andrews, 2023), and various non-routine mathematical problems (Arslan and Yazgan, 2015; Elia et al., 2009; Keleş and Yazgan, 2021). However, conflicting results exist in the literature (Elia et al., 2009; Hickendorff, 2018; Torbeyns et al., 2017; Xu et al., 2017). Our findings suggest that successful solving of non-routine problems requires strategic flexibility. Specifically, an increase in strategic flexibility is anticipated to enhance students’ ability to switch between various strategies and effectively implement them.

When examined by indicators, “selection of the appropriate strategy,” and “changing strategies when encountering different questions” are highly correlated with success in non-routine problem solving. Additionally, the indicators with the highest correlations with students’ strategic flexibility scores are “strategy knowledge,” “changing strategies when encountering different questions,” and “selection of the appropriate strategy”. These findings contribute to defining flexibility by many researchers, advocating for learning/applying multiple strategies, using multiple strategies, and making appropriate choices among strategies (Newton et al., 2020; Rittle-Johnson et al., 2012; Silla et al., 2024; Star, 2005; Star et al., 2022).

Our third research question examined which gender and strategic flexibility indicators among gifted fourth-grade students determine their success in solving non-routine problems. In the regression model created, the variable “selection of the appropriate strategy” from strategic flexibility indicators was found to be a significant predictor of students’ group membership. This study refines the understanding of strategic flexibility by empirically illustrating that among its multiple components, “selection of the appropriate strategy” emerges as the strongest predictor of non-routine problem-solving success in gifted fourth-grade students. According to the binary logistic regression equation established with this variable, the correct classification percentage is 93.9%. It was observed that the indicators “changing strategies when they do not work,” “solving the question again with a different strategy,” “using several strategies simultaneously,” “changing strategies when encountering different questions,” and the gender variable were not significant predictors of whether students had high or low levels of unusual problem solving success. This finding highlights the central role of strategic selection within the construct of flexibility and suggests that not all components of flexibility equally contribute to successful problem solving at this developmental stage. An increase of one unit in the “selection of the appropriate strategy” indicator is shown to increase the likelihood of students’ success in solving non-routine problems by 4560.7%. In other words, students who choose appropriate strategies have a higher probability of success. Thus, our findings contribute to refining the operational definition of strategic flexibility by identifying which specific dimensions have the most predictive validity in problem-solving contexts, particularly for gifted learners. Competence in selecting and using an appropriate mathematical strategy reflects students’ problem-solving performance levels (Cai, 2003). Stein et al. (2007) observed that the ability of students to reason through non-routine problems and choose appropriate problem solving strategies is crucial. Effective problem solving in mathematics hinges on the selection and application of suitable strategies (Star et al., 2022; Xu et al., 2017). Consequently, it is unsurprising that the ability to “selection of the appropriate strategy” correlates with higher problem solving success. This correlation is supported by research indicating that strategic selection enhances problem solving outcomes (Verschaffel et al., 1999). Scholars argue that flexibility in strategy selection involves making optimal choices among available methods for problem resolution (Newton et al., 2020; Rittle-Johnson et al., 2012; Star et al., 2022; Verschaffel et al., 2009). However, these findings highlight potential drawbacks of focusing solely on “selection of the appropriate strategy” in educational programs aimed at fostering strategic flexibility. Strategic flexibility encompasses not only the “selection of the appropriate strategy” but also multidimensional skills such as “changing strategies when they do not work” or “after solving the problem, solving it again with a different strategy”. Focusing exclusively on selecting the most accurate strategy in education may limit students’ flexible thinking and creative problem-solving abilities, leading to a one-dimensional development of their problem-solving skills. Therefore, programs that allow gifted students to develop all aspects of strategic flexibility in a balanced way will enable them to demonstrate flexibility across a wide range of problems and generate innovative solutions.

Regarding gender’s impact on non-routine problem solving success, the research suggests that gender does not significantly influence outcomes. This finding aligns with previous studies on gender differences in mathematical flexibility (Wang and Star, 2023). However, it contrasts with findings from Star et al. (2015), and Carr and Jessup (1997). The lack of gender differentiation in problem solving performance in this study could be attributed to all students being SAC students, undergoing identical intelligence testing, and following the same educational curriculum.

### 4.1 Educational implications

In addressing non-routine problems, employing and mastering multiple strategies can significantly enhance students’ ability to learn flexibly and experiment with novel approaches, thereby fostering creativity (Silla et al., 2024; Verschaffel et al., 2009; Xu et al., 2017). As Jiang et al. (2023) emphasize, students’ prior familiarity with different methods greatly influences their strategic choices. Consequently, classroom practices should not only include instruction in various strategies but also create opportunities for students to practice, reflect on, and compare different problem-solving approaches. Developing students’ strategic flexibility should thus be a central goal of curriculum and instruction.

However, many students perceive mathematics as a field of rigid procedures and single-solution answers, often associating success with rote memorization rather than adaptive thinking (Higgins, 1997; Schoenfeld, 1992). This perception may hinder the development of flexible problem-solving skills, particularly those such as checking the correctness of a solution with a different strategy or attempting multiple strategies for the same problem. To address this, classroom environments must intentionally emphasize and reward flexible thinking.

In this regard, several concrete strategies can be employed by educators. For instance, teachers can incorporate open-ended, non-routine problems that explicitly invite multiple solution paths and ask students to solve a problem in more than one way (Durkin et al., 2023; Star and Rittle-Johnson, 2008). Subsequent classroom discussions can focus on comparing different methods, evaluating their appropriateness, and reflecting on students’ choices (Newton et al., 2020; Rittle-Johnson et al., 2012). Teachers themselves can model metacognitive thinking by verbalizing their decision-making process during problem solving, thereby making strategic shifts and evaluations visible to students (Goos, 2002).

Moreover, establishing a classroom culture where errors are treated as learning opportunities and where verifying solutions through alternative methods is encouraged can help shift students’ epistemological beliefs about mathematics (Higgins, 1997; Schoenfeld, 1992). Finally, tools such as flexibility rubrics or strategy journals can be used to monitor and support students’ growth in flexible problem-solving behavior (Hong et al., 2023). These practices not only promote mathematical understanding but also nurture learners who are more adaptive, creative, and reflective—qualities essential for success in complex, real-world problem situations.

### 4.2 Limitations and suggestions for future studies

This study presents notable limitations. Primarily, the research was confined to a single SAC, located in Bursa, Türkiye. As such, the results may not be generalizable to all SACs or to broader populations of gifted students, particularly those in different geographic, socio-economic, or instructional contexts. The educational environment, teacher autonomy, and instructional strategies specific to this SAC may have influenced both the strategic flexibility and problem-solving performance of the participants. Future research should consider including multiple SACs across diverse regions and controlling for potential socio-economic or instructional variables to improve the external validity of the findings.

Future investigations could focus on longitudinally tracking the development of strategic flexibility in solving non-routine problems. Besides, further studies might include fourth-grade students who are not classified as gifted. Experimental research aimed at fostering strategic flexibility in problem solving through non-routine problems could also be pursued. Additionally, the exploration of strategic flexibility could encompass both algebraic problems (such as equation solving and operational adaptability) and other forms of non-routine problems.

## Data Availability

The raw data supporting the conclusions of this article will be made available by the authors, without undue reservation.

## Appendix: problems used in the study

P1) Half of half of half of a bag of apples equals 3 apples. How many apples are in the entire bag?
P2) An ice cream store has vanilla, cocoa, pistachio, lemon, and strawberry ice cream. How many different ways can you have two different scoops of ice cream in a cone?
P3) The tree in the sequence is fifth from both ends. How many trees are there in total in the sequence?
P4) A zookeeper has ostriches and elephants in one part of the zoo. Altogether the animals account for 60 heads and 180 legs. How many of each animal does he have?
P5) Using the clues given, determine which animal belongs to whom among Zeynep, Murat, and Batu:

- Zeynep’s animal has four legs.
- Murat’s animal does not bark.
- Batu is allergic to cats.
- Murat’s animal does not fly.
  P6) Melek is shorter than Burcu but taller than Ayşe. Elif is shorter than Ceren but taller than Burcu. Who is the tallest and who is the shortest of the 5 girls?
